# The use of 12-item General Health Questionnaire (GHQ-12) in Ukrainian refugees: translation and validation study of the Ukrainian version

**DOI:** 10.1186/s12955-024-02226-1

**Published:** 2024-01-13

**Authors:** Roberto Benoni, Anna Sartorello, Mariangela Mazzi, Loretta Berti, Marina Sorina, Elena Paiola, Giovanna Varischi, Stefano Tardivo, Michela Rimondini, Francesca Moretti

**Affiliations:** 1 Department of Diagnostics and Public Health, University of Verona, Verona, Italy; 2 Department of Neurosciences, Biomedicine and Movement Sciences, University of Verona, Verona, Italy; 3 “Malve di Ucraina” Non-profit Organization (NPO), Verona, Italy; 4 Prevention Department, Unità Locale Socio Sanitaria (ULSS) 9, Verona, Italy

**Keywords:** GHQ-12, Ukrainian refugees, Validation, Mental health, ITQ, Confirmatory factor analysis

## Abstract

Following the Russian-Ukrainian conflict, the well-being of millions of Ukrainians has been jeopardised. This study aims to translate and test the psychometric features of the Ukrainian version of the General Health Questionnaire 12 (GHQ-12). The study included Ukrainian refugees housed in Verona (Italy) between November/2022 and February/2023. The Ukrainian translation was obtained through a ‘forward-backward’ translation. Questionnaire was completed by 141 refugees (females: 78.7%). Median age was 36 years (IQR 23–43). Individuals with a score suggestive of psychological distress were 97 (68.8%). Cronbach’s coefficient was 0.84 (0.95CI 0.80–0.88). According to confirmatory factor analysis, both single- (modelB1) and two-factor (model B2) structures with bimodal scoring method fitted the data satisfactorily. The two factors of model B2 had a 0.88 correlation. Pearson coefficient showed a positive significant correlation between the GHQ-12 and International Trauma Questionnaire scores (ρ = 0.53, 0.95CI 0.40–0.64, *p* < 0.001). The GHQ-12 Ukrainian translation showed good psychometric features being a reliable and valid instrument to assess Ukrainian refugees’ general well-being.

**Supplementary Information:**

The online version contains supplementary material available at 10.1186/s12955-024-02226-1.

## Background

After the outbreak of the Russo-Ukrainian war on 24 February 2022, 8.2 million Ukrainians have been displaced or led to flee all over Europe, as of May 2023 [1]. Data on the consequences of the current war in Ukraine on the psychological well-being of refugees is still limited. Preliminary data on resettled Ukrainian refugees have only been reported from a study conducted in Germany. It found a prevalence rate of depressive and anxiety symptoms of 44.7% and 51.0%, respectively [2]. Refugee mental health assessment is particularly challenging since it may require cultural mediators and/or interpreters to facilitate communication and dialogue, and to fully understand the health status and the underlying needs. If the refugee feels that he/she is heard and understood, he/she may show an enhanced help-seeking behavior when in need [3]. This is even more important for mental health conditions, especially those at risk of self-harm and suicide.

In recent years, there was a growing awareness of the role of mental health in global health outcomes, premature deaths, and economic losses [4]. Research has shown that the prevalence of mental disorders, such as post-traumatic stress disorder (PTSD) and depression, is higher in the refugees than in the general population. A prevalence rate of 22.7%, 13.8%, and 15.8% for PTSD, depression, and anxiety disorders, respectively, was found in child and adolescents refugees resettled in Europe [5]. Risk factors for mental health are many and diverse and can change depending on the moment and the migration context. They can be distinguished into risk factors of the pre-migratory context, i.e. when the person is in the country of origin, where he or she may be directly or indirectly exposed to war and suffer trauma; during migration, as the journey itself may expose refugees to further traumatic events; and third, the post-migratory context may be a source of further stress for the refugee due to social isolation, unemployment and difficult cultural integration [6]. The importance of mental health and well-being as factors influencing the overall health status of refugees during migration and in the resettlement country has been widely recognized [5].

In order to assess the health status and needs of this vulnerable population, it is crucial to provide primary health workers with reliable and easy-to-use tools that allow a multicultural approach, such as short and simple questionnaires. These can reach large numbers of people and help health workers identify individuals at risk and provide timely assistance.

The General Health Questionnaire (GHQ) is a widely used assessment instrument of current psychological distress developed by Goldberg in 1970. In the following decades, different shortened versions of the original 60-items tool, such as the GHQ-30, GHQ-28, and the GHQ-12, have been proposed [7]. The questionnaire assesses the presence and severity of some psychological and psychosomatic symptoms over the previous few weeks using a self-reported four-point scale expressing whether a particular symptom or behaviour has recently been experienced by the respondent from less to much more than usual. The GHQ-12 most common scoring methods are bimodal (0–0–1-1) and Likert (0–1–2-3) resulting in a total score of 12 or 36 points, respectively [8]. The GHQ-12, due to its ease of use and brevity, has been extensively used to screen psychological distress in primary health care, outpatient settings, and in different cultures and populations [9, 10]. The GHQ-12 has also proved to be a consistent and reliable instrument when used in the refugee population [11]. Therefore, this study aims to translate the 12-item General Health Questionnaire (GHQ-12) into Ukrainian and to test its psychometric features (i.e. construct validity, internal consistency, and concurrent validity).

## Methods

### Ethical approval

The research was performed following the ethical standards of the 1964 Declaration of Helsinki and was approved by the Ethical Committee of the University Hospital of Verona on 24/10/2022 (protocol number 63939).

### Study design, setting, and population

This is a cross-sectional validation study. It was carried out in the province of Verona. The reception system in Italy for Ukrainian refugees is built on two different services provided by the governmental authorities, under the Home Office: the Reception and Integration System (RIS), managed at the local level and the Special Reception Centres (SRC), centrally managed [12]. Alongside these systems is the extended network of reception consisting of nonprofit organizations, social service centers, religious organizations, and co-housing measures with families or accommodation provided by other private entities. In Verona, the reception network supporting Ukrainian refugees is coordinated among all 98 municipalities in the province and includes about 117 SRC and four projects related to the RIS [13, 14]. As of April 2023, the number of Ukrainian refugees in the province of Verona reached 2265, of whom 1623 (71.7%) were females [15].

All persons who arrived in Italy from Ukraine after 24 February 2022, following the outbreak of the Russian-Ukrainian conflict, were considered eligible for this study. Refugees older than 14 years old whose native language was Ukrainian were included.

### Sample size

According to Mundfrom et colleagues [16] considering a ratio of variables to factors (p/f) of 6 and a two-factor solution, as in the original questionnaire [17], in a level of communality set as low, the minimum sample size to obtain an excellent-level criterion (0.98) was 120. Accounting for a drop-out rate of 15%, the target sample of participants was set at 146 for this study.

### Data collection

Data was collected between November and February 2023, progressively including all persons meeting the inclusion criteria until the computed sample size was reached.

Ukrainian refugees were recruited in the province of Verona through the local refugee reception network (i.e., regional and local authorities, SRC, RIS, and non-profit organizations).

A written disclosure about the study was first given and those who agreed to participate signed an informed consent form. Both documents were written in Ukrainian, the participants’ mother language. For those under the age of 18, informed consent was signed by their parents or legal guardian.

Each participant was asked to complete the Ukrainian translation of the GHQ-12 together with a short sociodemographic questionnaire (i.e., age, sex, education level, and marital status) and the subscale for PTSD of the International Trauma Questionnaire (ITQ) to serve as external validation. At all phases of the study, the research team was supported by a cultural mediator.

### Instruments

The original GHQ-12 consists of 12 items to be answered by the participant according to the variation, compared to his or her habitual standard, in the frequency of scenarios or behaviors described in the specific statement of the items (Table 1). The GHQ-12 has 6 positive items (answers options: “Better than usual”, “Same as usual”, “Less than usual”, “Much less than usual”) and 6 negative items (answers options: “Not at all”, “No more than usual”, “Rather more than usual”, “Much more than usual”).

**Table 1:** Original English and Ukrainian translation of the 12 items of the General Health Questionnaire 12 (GHQ-12). UKR: Ukrainian

Item	GHQ-12	UKR GHQ-12
q1	Been able to concentrate on what you’re doing?	Зосередитися на тому, що ви робите?
q2	Lost much sleep over worry?	Втратити сон через хвилювання?
q3	Felt you were playing a useful part in things?	Відчувати, що ви відіграєте корисну роль у справах?
q4	Felt capable of making decisions about things?	Відчувати себе здатними приймати рішення?
q5	Felt constantly under strain?	Постійно відчувати напругу?
q6	Felt you couldn’t overcome your difficulties?	Відчувати, що не можете подолати свої труднощі?
q7	Been able to enjoy your normal day-to-day activities?	Насолоджуватися своєю звичайною повсякденною діяльністю?
q8	Been able to face up to your problems?	Протистояти своїм проблемам?
q9	Been feeling unhappy and depressed?	Почуватися нещасними та пригніченими?
q10	Been losing confidence in yourself?	Втратити впевненість у собі?
q11	Been thinking of yourself as a worthless person?	Вважати себе нікчемною людиною?
q12	Been feeling reasonably happy, all things considered?	Почуватися досить щасливими, незважаючи на обставині?

In the present study, both scoring methods, bimodal and Likert, were evaluated. In the bimodal scoring method, the response categories have a score of 0, 0, 1, 1 for the positive items, while the negative items are scored the other way round (1,1,0,0). Therefore, the score ranges from 0 to 12 points. In the Likert scoring method, the positive items scored from 0 to 3 and the negative ones from 3 to 0, with a score range between 0 and 36 [18]. The most used cut-offs are between 2 and 4 for the bimodal method and ranged between 10 and 15 for the Likert one [18].

The ITQ is a self-report measure that allows a simple and concise assessment of key aspects of PTSD, according to the ICD-11 diagnostic criteria. The ITQ has two main subscales: the first (9 items), concerns PTSD and assesses three symptom domains, namely re-experiencing, avoidance, and sense of threat; the second (9 items), used to assess the complex PTSD, investigates the symptoms of self-organization disorder and the functional impairment caused by them. Each item is answered on a Likert scale from 0 (not at all) to 4 (very much). The cut-off for PTSD is given by a score > 2 in at least one of the two items of each of the three symptom domains (re-experiencing, items 1 and 2; avoidance, items 3 and 4; hyperarousal, items 5 and 6) plus at least one of the three indicators of functional impairment (items 7, 8 and 9). The ITQ is available in the Ukrainian language-validated version [19].

The PTSD subscale was used in the present study. Previous studies have analyzed psychological distress by combining the PTSD symptom score from the ITQ and the mental health problem risk score from the GHQ-12 to test the links between mental health, well-being, and conflict exposure [20].

### Translation and pilot testing

**Fig. 1:**
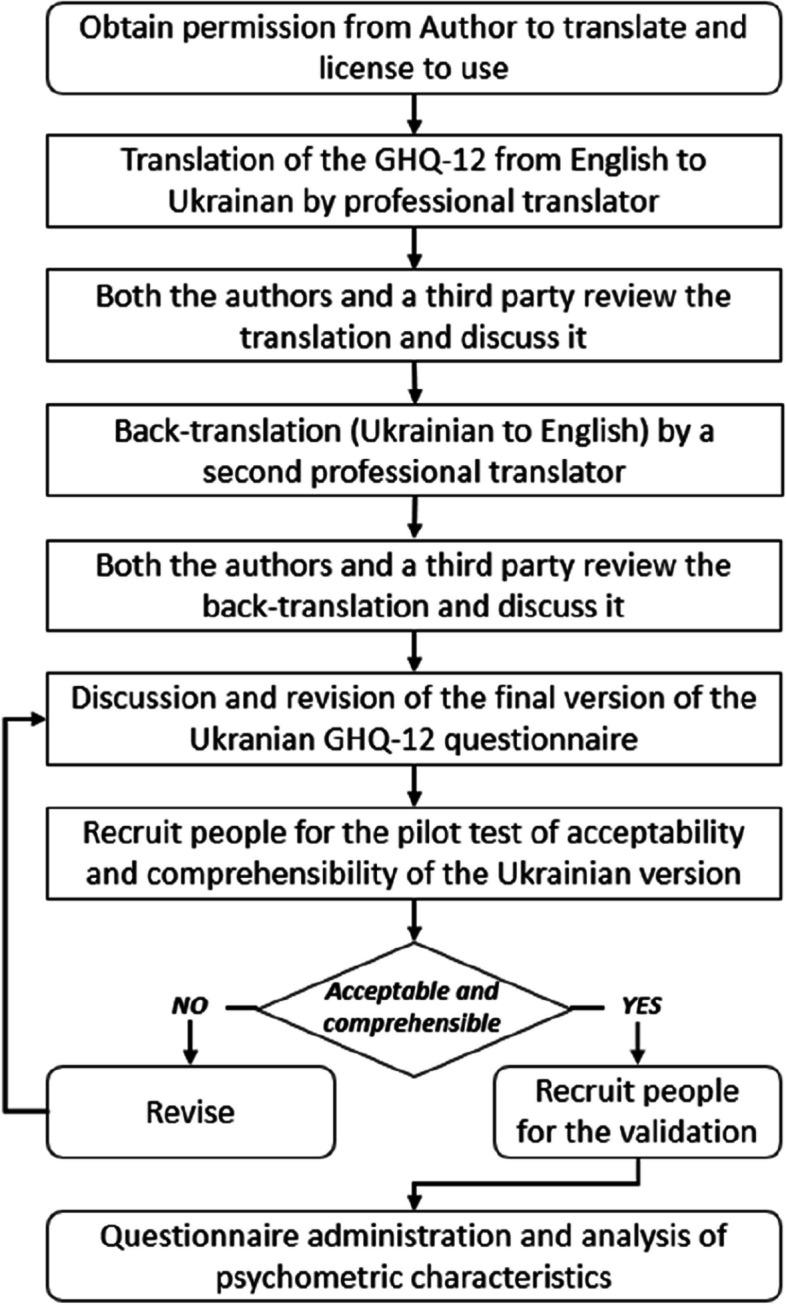
Flowchart of the translation, pilot test and validation process of the Ukrainian translation of the General Health Questionnaire 12 (GHQ-12) adopted in the present study

The translation process followed the WHO guidelines, which include a forward translation into the target language, i.e. Ukrainian, followed by a backward translation into the original language, i.e., English (Fig. 1) [21].

After obtaining permission from the Author to translate and the license to use the questionnaire, a professional translator provided the first Ukrainian version of the GHQ-12 from the original English questionnaire. This version was then revised with a third party fluent in both languages. The back-translation was carried out independently by a second professional translator who had not seen the original questionnaire in English. Both the authors and a third person reviewed the translation and revised it consensually. To avoid any conceptual losses during the translation process, the consensual retranslation was then compared with the original GHQ-12.

The translated questionnaire was initially administered to a sample of 28 refugees to test the acceptability and comprehensibility of the Ukrainian version. After completing the questionnaire, a cognitive interview was conducted to assess the clarity of the questions, any problems or difficulties in answering, and possible improvement actions. The pilot-sample was recruited based on sociodemographic criteria in order to be representative of both genders and different age groups (adolescents, adults, and elderly). Refugees who participated in the pre-test were not included in the final study sample.

The original English GHQ-12 and the Ukrainian GHQ-12 are available in the Supplementary material.

### Statistical analysis

A descriptive statistic was first conducted on sociodemographic data using frequencies and proportions for categorical variables and means and standard deviations (SD) or medians and interquartile ranges (IQRs) for continuous ones. Sample distribution was tested via χ2 and Fisher exact test or Mann-Whitney-U non-parametric, as appropriate.

GHQ-12 internal consistency was assessed through Cronbach’s alpha and McDonald’s omega coefficient testing the reliability and considering satisfactory a coefficient greater than 0.70. A tetrachoric correlation matrix was generated to assess the correlation between all the items of the GHQ-12 scored with a bimodal method.

A confirmatory factor analysis (CFA) was carried out to examine the factor structure of the Ukrainian version of the GHQ-12. First, a single-factor structure that contained all the GHQ-12 items was assessed. Secondly, a two-factor structure was tested encompassing two correlated latent factors: “Anxiety/Depression” (items: q1, q3, q4, q7, q8, q12) and “Social Dysfunction” (items: q2, q5, q6, q9, q10, q11). The two-factor structure was the one suggested by the author of the original English version of the GHQ-12 [16].

The models were tested for both the scoring method; for the bimodal method, the diagonally weighted least squares estimator was used and all variables were considered as ordered (ordinal) variables, for the Likert method, the maximum likelihood estimator was used with the Satorra-Bentler adjustment accounting for non-normality and heteroscedasticity of the data [22]. Model fit was evaluated using the χ2 test, the comparative fit index (CFI), the Tucker-Lewis index (TLI), the root-mean square error of approximation (RMSE), and the standardized root-mean-square residual (SRMR). Variance explained by latent variables was assessed through Average Variance Extracted (AVE). Criteria for acceptable model fit indices were based on Hooper et al. [23].

Pearson product moment statistic (Pearson’s correlation coefficient = *“ρ”*) was used to assess the concurrent validity of the GHQ-12 as the correlation with the ITQ subscale for PTSD. It was expected that the GHQ-12 would positively correlate with the ITQ subscale. A coefficient *“ρ”* above 0.40 was considered satisfactory. Association between single item score of the GHQ-12 and being screened positive for PTSD at the ITQ was conducted via z-test and t-test for bimodal and Likert scoring methods, respectively.

A *p*-value < 0.05 was considered significant. All analyses were performed using the R software (version 4.3.0).

## Results

### Sample characteristics

A total of 150 participants were recruited and 141 (94%) completed the questionnaire. The majority were females (*n* = 111, 78.7%), and the median age was 36 years (IQR 23–43). The level of education of the majority of the sample was university degree or higher (*n* = 77, 54.6%) followed by high school diploma (*n* = 32, 22.7%). Concerning marital status, 76 (53.9%) were married or in a de facto union, 39 (27.7%) were single, and 18 (12.8%) were divorced.

The mean score at GHQ-12 scored with the binomial method was 4.8 points (SD 3.4). Using two of the most used cut-offs in literature for the bimodal scoring method, i.e., > 3 and > 4, the percentage of people screened positive was 97 (68.8%) and 85 (60.3%), respectively. Those with a score equal to or higher to the mean GHQ-12 score for the whole study sample were 72 (51.1%). Table 2 shows descriptive statistics for the single items of the GHQ-12 based on both scoring methods (bimodal and Likert). The mean score at the ITQ subscale for PTSD was 14.0 points (SD 8.3). People with an ITQ score suggestive of PTSD were 59 (41.8%).

**Table 2:** Descriptive table of the total and single item scores in the General Health Questionnaire 12 (GHQ-12) based on the bimodal and the Likert scoring method and their association with the results at the International Trauma Questionnaire (ITQ) assessing the presence of post-traumatic stress disorder (PTSD)

	GHQ-12 bimodal scoring				GHQ-12 Likert score			
Item	% score = 1 (overall)	% score = 1 (ptsd = 1)	% score = 1 (ptsd = 0)	*p*-Value*	mean (sd) (overall)	mean (sd) (ptsd = 1)	mean (sd) (ptsd = 0)	*p*-Value*
q1	39.0%	55.9%	26.8%	< 0.001	1.40 (0.88)	1.66 (0.96)	1.21 (0.77)	< 0.001
q2	55.3%	71.2%	43.9%	0.001	1.55 (1.09)	1.95 (1.01)	1.26 (1.05)	< 0.001
q3	39.0%	45.8%	34.1%	0.160	1.35 (0.86)	1.47 (1.02)	1.26 (0.72)	< 0.001
q4	22.7%	30.5%	17.1%	0.060	0.99 (0.80)	1.05 (0.95)	0.95 (0.66)	< 0.001
q5	66.0%	83.1%	53.7%	< 0.001	1.82 (0.87)	2.12 (0.77)	1.60 (0.87)	< 0.001
q6	48.2%	61.0%	39.0%	0.010	1.46 (0.95)	1.69 (0.90)	1.29 (0.95)	< 0.001
q7	52.5%	74.6%	36.6%	< 0.001	1.65 (0.85)	2.05 (0.84)	1.35 (0.74)	< 0.001
q8	28.4%	35.6%	23.2%	0.107	1.13 (0.84)	1.22 (1.00)	1.06 (0.71)	< 0.001
q9	46.8%	55.9%	40.2%	0.065	1.33 (0.97)	1.46 (1.07)	1.23 (0.88)	< 0.001
q10	33.3%	47.5%	23.2%	0.003	1.02 (0.98)	1.25 (1.06)	0.85 (0.89)	< 0.001
q11	10.6%	16.9%	6.1%	0.040	0.43 (0.76)	0.56 (0.93)	0.34 (0.59)	0.853
q12	41.1%	57.6%	29.3%	< 0.001	1.36 (0.92)	1.63 (1.02)	1.17 (0.80)	< 0.001
**Total**								
Mean (sd)	4.8 (3.4)	6.4 (3.2)	3.7 (3.1)	< 0.001	15.5 (6.8)	18.1 (7.2)	13.6 (5.9)	< 0.001

*Z-test, *T* test, *sd* standard deviation

### Concurrent validity

Validity was assessed through Pearson correlation coefficient between the total score at GHQ-12 and the ITQ subscale for PTSD. A positive significant correlation was found with a coefficient *“ρ”* equal to 0.53 (0.95CI 0.40–0.64, *p* < 0.001). When looking at the association between the single items and a suggestive score for PTSD at the ITQ, eight items showed a positive significant association (Table 2). The items more frequently associated with PTSD and with the highest difference between positive and negative PTSD proportions were item 7 (74.6%), item 5 (83.1%), and item 1 (55.9%).

### Construct validity

**Fig. 2:**
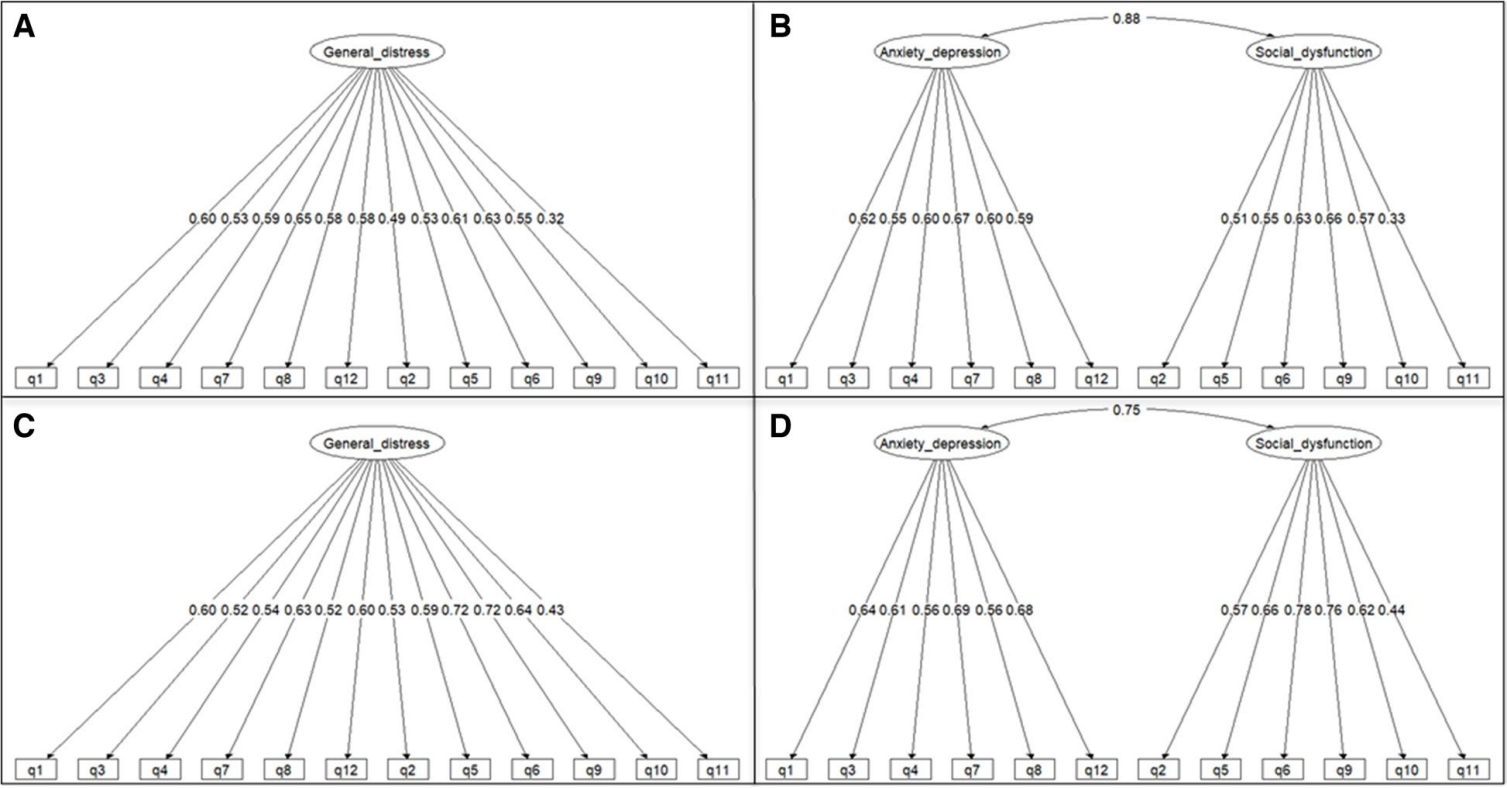
Standardized parameter estimates from the models fitted on single- and two-factor structure with bimodal scoring method (panels top left and top right) and with Likert scoring method (panels bottom left and bottom right)

The results of the CFA are shown in Table 3. The bimodal scoring method had good indices for both single- (model B1, TLI = 0.98, RMSEA = 0.05[0.90CI 0.00–0.07]) and two-factor models (model B2, TLI = 0.98, RMSEA = 0.04[0.90CI 0.00–0.07]). In model B2 the two subscales had a high correlation index, equal to 0.88. Both B1 and B2 models achieved a satisfactory AVE above 0.50. In the Likert scoring method, the single factor model (model L1) didn’t fit the data well (TLI = 0.77, RMSEA = 0.11[0.90CI 0.09–0.13]). The two-factor model (model L2) showed better and acceptable indices (TLI = 0.58, RMSEA = 0.09[0.90CI 0.06–0.11]). Model L2 had a correlation of 0.75 between the two subscales. Figure 2 shows the standardized parameter estimates for all the four models.

**Table 3:** Confirmatory factor analysis of Ukrainian version of the General Health Questionnaire 12. Model fit statistics for single-factor structure (models B1 and L1) and two-factor structure (models B2 and L2) for the bimodal and Likert scoring methods, respectively

	Χ2	Df	p-Value	CFI	TLI	RMSEA	0.90CI	SRMR	AVE
**Bimodal**									
**Model B1**	63.21	54	0.080	0.981	0.976	0.045	0.000–0.073	0.097	0.521
**Model B2**	64.41	53	0.135	0.985	0.982	0.039	0.000–0.070	0.093	f1 = 0.581 f2 = 0.520
**Likert**									
**Model L1**	144.69	54	< 0.001	0.803	0.766	0.109	0.089–0.129	0.085	0.358
**Model L2**	108.14	53	< 0.001	0.880	0.851	0.086	0.064–0.107	0.073	f1 = 0.394 f2 = 0.429

*Df* degrees of freedom: *CFI* comparative fit index: *TLI* Tucker-Lewis index: *RMSEA* root-mean square error of approximation: *SRMR* standardized root-mean-square residual: *AVE* Average Variance Extracted: *f1* Anxiety and depression: *f2* Social dysfunction: *f3* Loss of confidence

### Internal consistency

The mean score of the GHQ-12 items was 0.40 (SD = 0.29). The items with the highest frequency of positive results (i.e., a score equal to 1) were item 5 (66%), item 2 (55%), and item 7 (53%) (Table 2). Reliability was tested with Cronbach’s and McDonald’s omega coefficients that were found to be 0.84 (0.95CI 0.80–0.88) and 0.85 (0.95CI 0.81–0.88) in the whole sample, respectively. The alpha and omega coefficients in the two subscales were 0.78 [0.95CI 0.71–0.83] and 0.78 [0095CI 0.72–0.83] for ‘anxiety/depression’ and 0.72 [0.95CI 0.64–0.79] and 0.73 [0.95CI 0.66–0.79] for ‘social dysfunction’. Stratifying by sex both alpha and omega coefficients remained consistent as in the whole sample (alpha: female = 0.84[0.95CI 0.80–0.88], male = 0.85[0.95CI 0.76–0.92]; omega: female = 0.84[0.95CI 0.75–0.92], male = 0.84[0.95CI 0.79–0.88]).

**Fig. 3:**
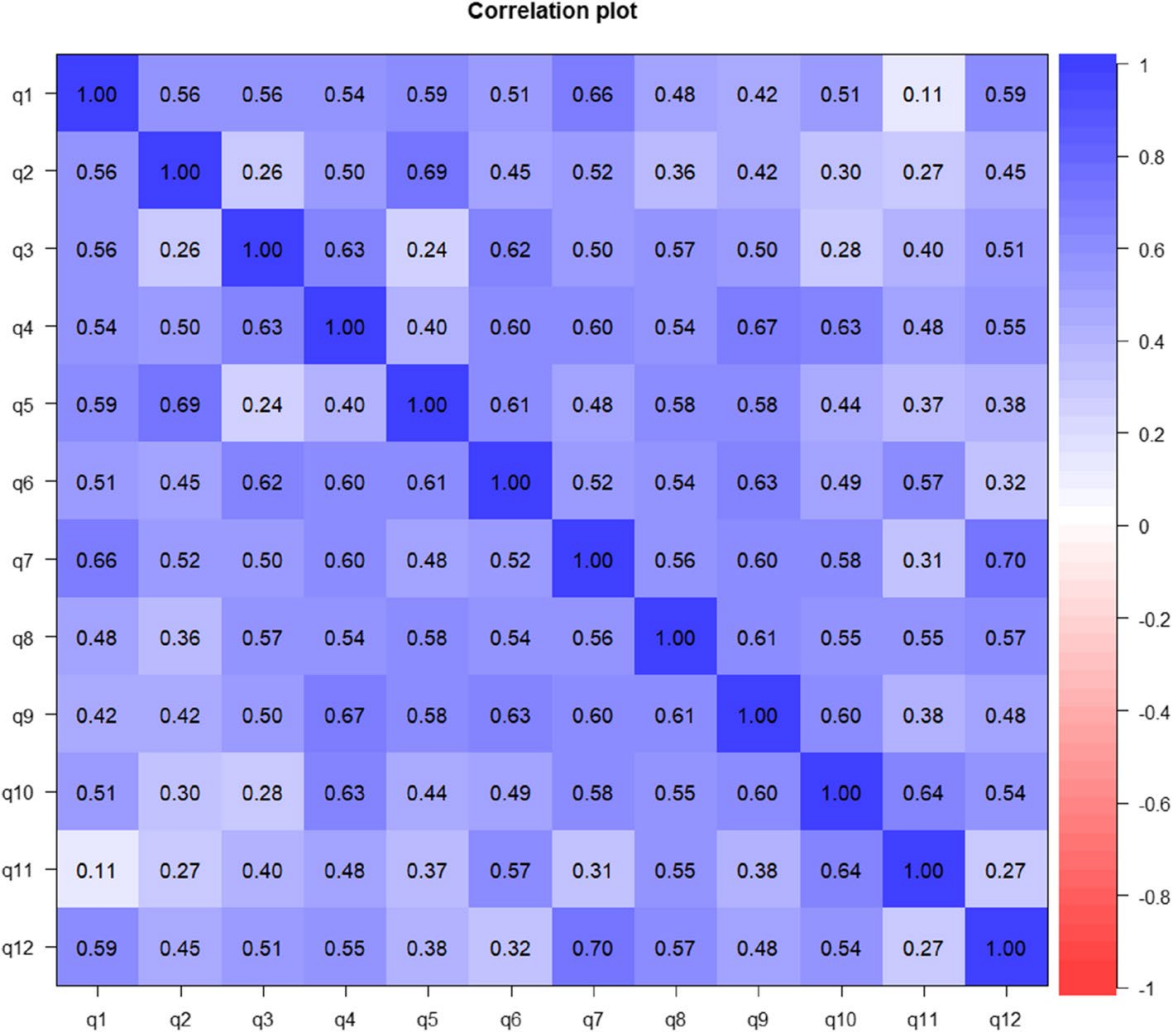
Tetrachoric correlation matrix of the Ukrainian version of the General Heath Questionnaire-12 with bimodal scoring method

The items with the highest correlation were q7 and q12 (0.695[0.95CI 0.502;0.835]), while those with the lowest were q 1 and q11 (0.114[0.95CI -0.242;0.468) (Fig. 3).

## Discussion

The present study showed that the Ukrainian translation of GHQ-12 had good reliability and validity and a two-factor structure consistent with the original English version.

The GHQ-12 is a well-known instrument to assess the general well-being and mental health, used in different populations and settings, including low- and middle-income countries [10]. It was widely used in several study designs (cross-sectional, RCT, and longitudinal) among migrants and refugees to screen for mental health disorders [24–26].

Internal reliability of the Ukrainian translation of the GHQ-12 was overall satisfactory in our study (alpha = 0.84). The Ukrainian GHQ-12 also showed a good level of concurrent validity through the correlation with the ITQ (ρ = 0.53). The GHQ-12 has previously been used with satisfactory results for screening refugees for PTSD [27]. This mental disorder is one of those that most affect refugees and one of the main ones examined in the literature on this population [28]. PTSD seriously endangers both the mental and general health of persons, as it can lead to self-harm and suicidal ideation and attempts. Only one study has previously used the GHQ-12 in Ukrainian refugees, although it only evaluated its internal reliability, finding an alpha of 0.83, as in the present study. It didn’t explore the validity and factorial structure of the Ukrainian translation of the GHQ-12 [2].

In the confirmatory factor analysis, both single- (model B1) and two-factor (model B2) structures with bimodal scoring methods fitted data well. The bimodal scoring system has previously proven its validity as a screening tool, as in the case of the present study, whereas the Likert method may be more useful for the follow-up of patients over time [29]. The GHQ-12 was originally developed as a unitary screening measure and the high correlation found in our sample between the two subscales in model B2 and L2 supports this structure. Several multidimensional factor constructions comprising two to three factors have been proposed and tested [30]. A multicentric study of psychological disorders in general health by WHO found a substantial factor variation between the 15 centres involved. However, after rotation two factors expressing “Anxiety/Depression” and “Social Dysfunction” were found for the GHQ-12 [17]. Another study comparing different factorial structures for the GHQ-12 found that a unidimensional model, with a general factor representing the commonality between all items and two orthogonal specific factors reflecting the common variance due to wording effects (negatively and positively worded items) and representing the two previously identified factors, was the best fit [31]. The present study showed that the Ukrainian translation of GHQ-12 is consistent with the factor structures proposed in the literature and very similar to that of the original English version.

Using a binary scoring method, as the original Goldberg version of the GHQ-12, we found a mean score of 4.8 points. Different cut-offs have been proposed in the literature depending on the population involved, mainly ranging between 2 and 4 [18]. As a rule of thumb, it has been proposed to use the mean score for the overall population of respondents as a rough guide to the best threshold [32]. The cut-off of screening tools is also driven by the prevalence of a specific disorder in a given population [32]. In the present study, the sample consisted of Ukrainian refugees. This is a well-known at-risk population for mental health disorders, and we therefore found a higher threshold than that proposed in the literature. Adopting a 5-point cut-off, 51% of the sample showed a suggestive score for mental distress. The GHQ, even in its short 12-item form, is therefore a robust self-report tool for screening people who may be at risk for mental health disorders, especially adolescent and young people [33]. For this reason, it could be particularly useful in the Ukrainian refugee population, made up mainly of young women and children. Simple tools to investigate the prevalence of people at risk of mental health problems are widely used such as the Refugee Health Screener-15 (RHS-15) as a general measure of emotional distress and the Primary Care PTSD Screen for DSM-5 (PC-PTSD-5). They have the advantage of being rapid and easy to be administered, allowing even non-specialized personnel to use them [34]. These questionnaires were used in a school setting to screen Ukrainian refugee adolescents, finding a prevalence of 57.1% and 45.2% above the critical cut-off of RHS-15 and PC-PTSD-5, respectively [35]. The GHQ in its short 12-item form can therefore complement these instruments and be used not only by clinicians but also by schools, nonprofit organizations, or social service personnel as a self-report tool to identify persons at risk for mental health at an early stage and to provide them with timely assistance and support.

This study has some limitations. First of all, it was conducted only in the province of Verona, so it may not be representative of the entire population of Ukrainian refugees. Likewise, it involved a particularly high-risk category, so it may not be generalizable to the entire Ukrainian population. Our sample was also unbalanced between males and females, with the latter being the most represented. This sample however reflects the composition of the study population. It would be useful to repeat this in a larger and more general sample of people in Ukraine to see if the results are confirmed. Moreover, a larger sample would have offered the possibility of conducting an analysis based on the item response theory to assess the invariance of the results concerning the characteristics of the participants. Secondly, the validation was assessed on a specific mental disorder, and this could be restrictive compared to the general health explored by the GHQ-12. Future studies, across different regions, should explore how the different cultural contexts may influence the responses and thus the validation of the questionnaire. Furthermore, the use of emerging techniques, such as clinimetric analysis, would be important to apply to verify the clinical properties of the Ukrainian version of the GHQ-12 [36].

## Conclusions

The present study showed that the Ukrainian translation of the GHQ-12 had good internal reliability and concurrent validity and showed a factor structure consistent with the original version. It provides a useful tool for assessing general well-being in an at-risk population such as Ukrainian refugees. To the best of our knowledge, this is the first study to provide a comprehensive validation of the Ukrainian translation of the GHQ-12. Future studies may use it on larger population samples both as a screening tool and to study factors associated with general and mental well-being in the resettlement country to improve reception and integration services for this vulnerable population.

### Supplementary Information

**Additional file 1.**

## Data Availability

The datasets generated and/or analysed during the current study are available from the corresponding author upon reasonable request.

## Abbreviations

CFA: Confirmatory Factor Analysis
GHQ: General Health Questionnaire
IQR: Inter Quartile Range
ITQ: International Trauma Questionnaire
PTSD: Post-Traumatic Stress Disorder
SD: Standard Deviation
WHO: World Health Organization
