# Supplementary material for: The use of 12-item General Health Questionnaire (GHQ-12) in Ukrainian refugees: translation and validation study of the Ukrainian version
Source: Health Qual Life Outcomes. 2024 Jan 13;22:6. doi: 10.1186/s12955-024-02226-1 (PMC10788012; doi:10.1186/s12955-024-02226-1)
Supplement: Supplementary file 1 — Additional file 1. [file 12955_2024_2226_MOESM1_ESM.pdf]

Supplementary material

Short General Health Questionnaire (GHQ 12)

Have you recently?

1. Been able to concentrate on what you're doing?	Better than usual	Same as usual	Less than usual	Much less than usual
2. Lost much sleep over worry?	Not at all	No more than usual	Rather more than usual	Much more than usual
3. Felt you were playing a useful part in things?	More so than usual	Same as usual	Less useful than usual	Much less useful
4. Felt capable of making decisions about things?	More so than usual	Same as usual	Less so than usual	Much less capable
5. Felt constantly under strain?	Not at all	No more than usual	Rather more than usual	Much more than usual
6. Felt you couldn't overcome your difficulties?	Not at all	No more than usual	Rather more than usual	Much more than usual
7. Been able to enjoy your normal day-to-day activities?	More so than usual	Same as usual	Less so than usual	Much less than usual
8. Been able to face up to your problems?	More so than usual	Same as usual	Less so than usual	Much less able
9. Been feeling unhappy and depressed?	Not at all	No more than usual	Rather more than usual	Much more than usual
10. Been losing confidence in yourself?	Not at all	No more than usual	Rather more than usual	Much more than usual
11. Been thinking of yourself as a worthless person?	Not at all	No more than usual	Rather more than usual	Much more than usual
12. Been feeling reasonably happy, all things considered	More so than usual	About same as usual	Less so than usual	Much less than usual

Дайте відповідь на наступні питання, думаючи про те, наскільки це описує Вас

Чи траплялось вам нещодавно…

**1**. Зосередитися на тому, що ви робите?	☐ краще, ніж звичайно	☐ як звичайно	☐ менш, ніж звичайно	☐ набагато менш, ніж звичайно
**2**. Втратити сон через хвилювання?	☐зовсім ні	☐ не більш ніж звичайно	☐ трішки більш, ніж звичайно	☐ значно більш, ніж звичайно
**3**. Відчувати, що ви відіграєте корисну роль у справах?	☐ більш, ніж звичайно	☐ як звичайно	☐ менш, ніж звичайно	☐ набагато менш, ніж звичайно
**4**. Відчувати себе здатними приймати рішення?	☐ більш, ніж звичайно	☐ як звичайно	☐ менш, ніж звичайно	☐ набагато менш, ніж звичайно
**5**. Постійно відчувати напругу?	☐ зовсім ні	☐ не більш ніж звичайно	☐ трішки більш, ніж звичайно	☐ значно більш, ніж звичайно
**6**. Відчувати, що не можете подолати свої труднощі?	☐ зовсім ні	☐ не більш ніж звичайно	☐ трішки більш, ніж звичайно	☐ значно більш, ніж звичайно
**7**. Насолоджуватися своєю звичайною повсякденною діяльністю?	☐ більш, ніж звичайно	☐ як звичайно	☐ менш, ніж звичайно	☐ набагато менш, ніж звичайно
**8**. Протистояти своїм проблемам?	☐ більш, ніж звичайно	☐ як звичайно	☐ менш, ніж звичайно	☐ набагато менш, ніж звичайно
**9**. Почуватися нещасними та пригніченими?	☐ зовсім ні	☐ не більш ніж звичайно	☐ трішки більш, ніж звичайно	☐ значно більш, ніж звичайно
**10**. Втратити впевненість у собі?	☐ зовсім ні	☐ не більш ніж звичайно	☐ трішки більш, ніж звичайно	☐ значно більш, ніж звичайно
**11**. Вважати себе нікчемною людиною?	☐ зовсім ні	☐ не більш ніж звичайно	☐ трішки більш, ніж звичайно	☐ значно більш, ніж звичайно
**12**. Почуватися досить щасливими, незважаючи на обставині.	☐ більш, ніж звичайно	☐ як звичайно	☐ менш, ніж звичайно	☐ набагато менш, ніж звичайно
